# Pre-treatment drug resistance among patients initiating antiretroviral therapy (ART) in Zimbabwe: 2008–2010

**DOI:** 10.1186/s13104-016-2101-8

**Published:** 2016-06-10

**Authors:** More Mungati, Mutsa Mhangara, Elizabeth Gonese, Owen Mugurungi, Janet Dzangare, Stella Ngwende, Patience Musasa, Maureen Wellington, Gerald Shambira, Tsitsilina Apollo, Chunfu Yang, Joshua DeVos, Jennifer Sabatier, Peter Kilmarx, Christine Chakanyuka-Musanhu, Mufuta Tshimanga

**Affiliations:** Ministry of Health and Child Care, Harare, Zimbabwe; Centers for Disease Control and Prevention, Harare, Zimbabwe; World Health Organization, Harare, Zimbabwe; Department of Community Medicine, University of Zimbabwe, Harare, Zimbabwe; National Microbiology Reference Laboratory (NMRL), Harare, Zimbabwe; Center for Disease Control and Prevention, Atlanta, GA USA; Newlands Clinic, Harare, Zimbabwe

**Keywords:** Prevalence, HIVDR, PDR, Surveillance, Genotyping

## Abstract

**Background:**

Zimbabwe set up 12 sentinel sites to monitor HIV drug resistance (HIVDR) following the international standards for prevention of HIVDR from 2008 to 2010.

**Methods:**

Participants were consecutively enrolled. Blood was collected and used for CD4 count, viral load (VL) and pre-treatment DR (PDR) tests besides routine baseline tests. We analyzed the characteristics of participants enrolled into the survey and estimated the point prevalence of PDR and its associated factors among ART initiators in a cross-sectional analysis using the baseline data collected from a prospective cohort in 12 purposefully selected sentinel sites.

**Results:**

A total of 1728 participants (96 % response rate) were enrolled and 1610 had complete data. Of the 1610 there were more females (68.7 %) than males (31.3 %). The median CD4 count was 168 cells/mm^3^ with males having lower values (*P* = 0.003). Ninety-six percent of participants had a VL ≥ 1000 copies/ml and the median VL was 128,000. Previous exposure to antiretroviral drugs (ARVs) was mainly through PMTCT (5 % of the participants). Overall, PDR mutations were detected in 6.3 % (95 % CI 5.2–7.7) of the 1480 successfully genotyped participants. However, the prevalence of PDR mutations was double for those with previous exposure (12.1 %) to ARVs compared with those without previous exposure (5.7 %, *P* = 0.002).

**Conclusions:**

The results show a moderate level of PDR prevalence among ART initiators. To maintain the efficacy of the current first-line regimens, there is need to strengthen all HIVDR prevention efforts and to conduct further studies to investigate optimal strategies that can prolong the efficacy of first-line ARV regimens in the country.

## Background

As the global scale-up of antiretroviral therapy (ART) increases there are growing concerns over the increase in the prevalence of human immunodeficiency virus (HIV) drug resistance (HIVDR). This is inevitable because of HIV’s rapid and error-prone replication, a high mutation rate in the presence of drug selective pressure, sub-optimal therapy and/or poor adherence to treatment schedules, and viral recombination [1]. HIVDR mutations may also be transmitted to ART naïve patients from HIV-infected patients who have HIV drug resistant viruses. Thus, some degree of HIVDR should be anticipated in patients starting first-line ART. Factors contributing to the development of HIVDR are regimen- or drug-specific, virus-related, patient-specific and programmatic factors [2, 3]. The provision of high-quality care and treatment, early diagnosis, and consistent follow-up are necessary to minimize the emergence of drug-resistant HIV.

In response to the concerns over patients in resource-limited countries developing HIVDR, the World Health Organization (WHO) developed a global HIVDR prevention strategy. This strategy recommends countries to conduct surveys that focus on identifying program and site-specific factors associated with HIVDR development, thus enabling site and program interventions to alter these factors. This HIVDR prevention strategy helps to maximize the long-term efficacy and durability of available antiretroviral regimens [4].

Routine testing of transmitted HIVDR is an important strategy in monitoring HIVDR in resource-rich countries to ensure the HIV-positive patients are prescribed the efficacious ART regimens [5]. Unfortunately, this is not available for most patients in resource-limited countries. Thus addressing this issue using public health approach has become a mammoth task to ensure the efficacy of the current first-line regimens in these countries.

In support of the ART scaling up and prevention of HIVDR emergence and transmission, Zimbabwe established 12 sentinel ART sites to monitor the prevalence of HIVDR in the period of 2008–2010. The aim of our surveys was to establish the prevalence and distribution of HIVDR, and to identify risk factors associated with HIVDR emergence. These surveys are critical to the Zimbabwe ART program in providing information on the appropriateness of the non-nucleoside reverse transcriptase inhibitors (NNRTI) and nucleoside reverse transcriptase inhibitors (NRTI) based first-line regimens recommended by WHO and interventions for preserving these affordable regimens for the years to come. In the current report, we analyzed the characteristics of participants enrolled into the surveys, and estimated the point prevalence of Pre-treatment DR (PDR) and its associated factors among ART initiators in a cross-sectional analysis using the baseline data collected from a prospective cohort in 12 purposefully selected sentinel sites.

## Methods

### ART site selection and participant enrolment

The ART sites were purposefully selected in a phased approach (three sites in 2008, five sites in 2009 and four sites in 2010) following the WHO-recommended methods [6], for a total of 12 sites. After signing the volunteer participation consent, participants were recruited sequentially as they initiated ART until the pre-determined sample size of 150 was reached at each site.

### Demographic and clinical data collection

Baseline patient socio-demographic and clinical data were obtained using a standard interviewer-administered questionnaire. Clinical data were assessed for correctness and accuracy by the HIVDR technical working group while at the same time support visits were provided to the sites to verify data submissions.

### Specimen collection

Ten millilitres of blood were collected from each participant using two ethylene-diamine-tetra-acetic acid (EDTA) tubes. One tube was used for routine baseline liver function, full blood and CD4 cell count tests and preparation of dried blood spot (DBS) specimens for genotyping on-site. Each participant had two DBS cards prepared (One was prepared for backup purpose) and each card had five spots containing 50 µl of blood on each spot. These DBS specimens were dried overnight, packaged following standard operating procedures (SOPs) and transported to the National Microbiology Reference Laboratory (NMRL) at ambient temperature where they were stored at −80 °C. The other tube was centrifuged on site to obtain plasma which was then aliquoted for viral load (VL) testing and HIVDR genotyping.

### Viral load measurement and HIVDR genotyping

Viral load testing was performed at NMRL using the Roche COBAS Taqman HIV-1 version 2 test with High Pure extraction system following the manufacturer`s instructions. An aliquot of plasma/DBS from each participant was shipped on dry ice to the WHO-designated DR laboratories, including the national DR laboratory at Medical Research Council of Uganda, Entebbe, Uganda; the National Institute of Communicable Diseases (NICD), South Africa and the specialized DR laboratory at CDC, Atlanta, United States for testing using validated and quality-assured in-house genotyping methods [7–9]. Genotyping analyses included both the protease (Pro) and reverse transcriptase (RT) gene regions and data quality was ensured by following SOPs established in the laboratories which include phylogenetic analysis to eliminate cross-contaminations. The quality-assured consensus sequences generated were then used to identify DR mutations interpreted using the Stanford HIV Drug Resistance Interpretations Algorithm (http://hivdb.stanford.edu/). The newly obtained HIV-1 pol sequences are submitted in the GeneBank and with accession numbers pending.

### Statistical analyses

Prevalence, frequencies, proportions, odds ratios, stratified analysis and Chi square tests were calculated using Epi info 3.5.4 (CDC, Atlanta, 2013) and Microsoft Excel ^®^2007 was used to draw graphs. All calculations were made at 95 % confidence intervals (CI). Univariate analysis was initially done fitting information into previously prepared shell tables and graphs. This was followed by bivariate analysis to examine two variables in association with risk factors and outcomes. Stratified analysis was done for factors found to be statistically significant in the bivariate analysis to control for confounding and identifying effect modification. The analysis was completed in Stata v13, with svyset commands to apply inverse probability weights that account for oversampling of provincial PSUs, and to adjust for clustering of observations within PSUs and stratification by site.

## Results

### Site characteristics, and participant demographic and clinical characteristics

Participants were enrolled from a Central Hospital, Provincial Hospital, Private Clinic, District Hospitals, Mission Hospital, and a Local Authority Clinic. All 12 sites were offering comprehensive Opportunistic Infection (OI)/ART services. Pre-study site assessment had showed that none of the 12 sites had adequate staff based on the Zimbabwe OI/ART Standard Operating Procedures (December 2006), although they all had the core staff for the purpose of initiating and managing participants on ART. There were no reported drug stock-outs within 6 months prior to the survey period. Ninety-six percent (1728) of the expected 1800 participants were enrolled at baseline with a median site enrolment of 97 %.

The 93.2 % (1610) of enrolled participants had complete data which were used for the analysis. Females constituted 68.7 % (1106) of all participants. Of the 87 reporting previous exposure to ARVs, 72.4 % (63) were women who had used single-dose nevirapine (NVP) for prevention of mother-to-child transmission of HIV (PMTCT). Most (70.8 %) participants had no previous exposure to ARVs while 5 % had known exposure status, 23.9 % had missing information on exposure and 0.4 % were classified as other.

Median CD4 count was 168 cells/mm^3^ with males having lower CD4 count than females (*P* = 0.003). Median VL was 128,000 copies/ml with no significant difference between males and females (*P* = 0.724) (Table 1). Table 1 highlights the WHO clinical staging for the 1610 participants (63.9 % were eligible for ART). Fifteen percent of participants had a CD4 count less than 100 cells/mm^3^. Of all participants enrolled and commenced on ART, 53.6 % were eligible for ART based on a CD4 count <350 cells/mm^3^ alone according to the National ART guidelines in place during the period of the study. Males were more likely to have advanced HIV infection (CD4 count <200) at ART initiation than females (OR 1.59; 95 % CI 1.22–2.07).

**Table 1:** Characteristics of the 1610 participants in the study among the 12 sentinel ART sites in Zimbabwe between 2008 and 2010

Characteristic	N (%)
*Gender*	
Female	1106 (68.7)
Male	487 (30.3)
Missing data	17 (1.1)
*Age*	
Median age (IQR)	36 (30;44)
*WHO clinical staging*	
Stage 1	106 (6.6)
Stage 2	289 (18.0)
Stage 3	951 (59.1)
Stage 4	78 (4.8)
Missing data	186 (11.5)
*Previous Antiretroviral drugs (ARVs) exposure*	
PMTCT	63 (3.9)
ARVs prior to initiation	18 (1.1)
Others	6 (0.4)
No exposure	1139 (70.7)
Missing exposure status	384 (23.9)
*CD4 count*	
Median baseline CD4 count (IQR)	168 (94;253)
Females	180 (106;265)
Males	136 (71;222)
*Viral loads (copies/ml)*	
≥1000	1417 (95.67)
Females	982 (66.31)
Males	435 (29.36)
<1000	46 (4.33)
*Drug resistance mutations*	
No. with genotyping results	1483
No resistance	1389
NNRTI resistance only	73 (4.9)
NRTI resistance only	18 (1.2)
NRTI + NNRTI resistance	5 (0.3)
PI resistance	10 (0.7)
Any resistance mutation	94 (6.3)

We also analyzed ART initiation criteria based on the WHO clinical stages in comparison to CD4 count. There were 515 patients in the WHO stage 3 or 4 with a CD4 count <350 cell/mm^3^, 514 with CD4 count <350 cells/mm^3^ in the WHO stage 1 or 2, 100 in the WHO stage 3 or 4 with CD4 count >350 cells/mm^3^, and 294 patients in the WHO stage 1 or 2 with CD4 count >350. According to the 2010 Zimbabwe National ART guidelines 294 patients were not eligible for initiation of ART based on CD4 count or WHO clinical staging.

### HIV-1 viral load determination and drug resistance genotyping

Among the 1610 participants who had completed dataset, VL data were available for 1463 (90.9 %) participants while 147 (9.1 %) were missing. Among the 1463 participants with VL measurements, 1417 (95.7 %) had VL levels greater than or equal to 1000 copies/ml while 46 (4.3 %) were less than 1000 copies/ml. There was no difference on VL levels between men and women (*P* = 0.72) (Table 1).

**Fig. 1:**
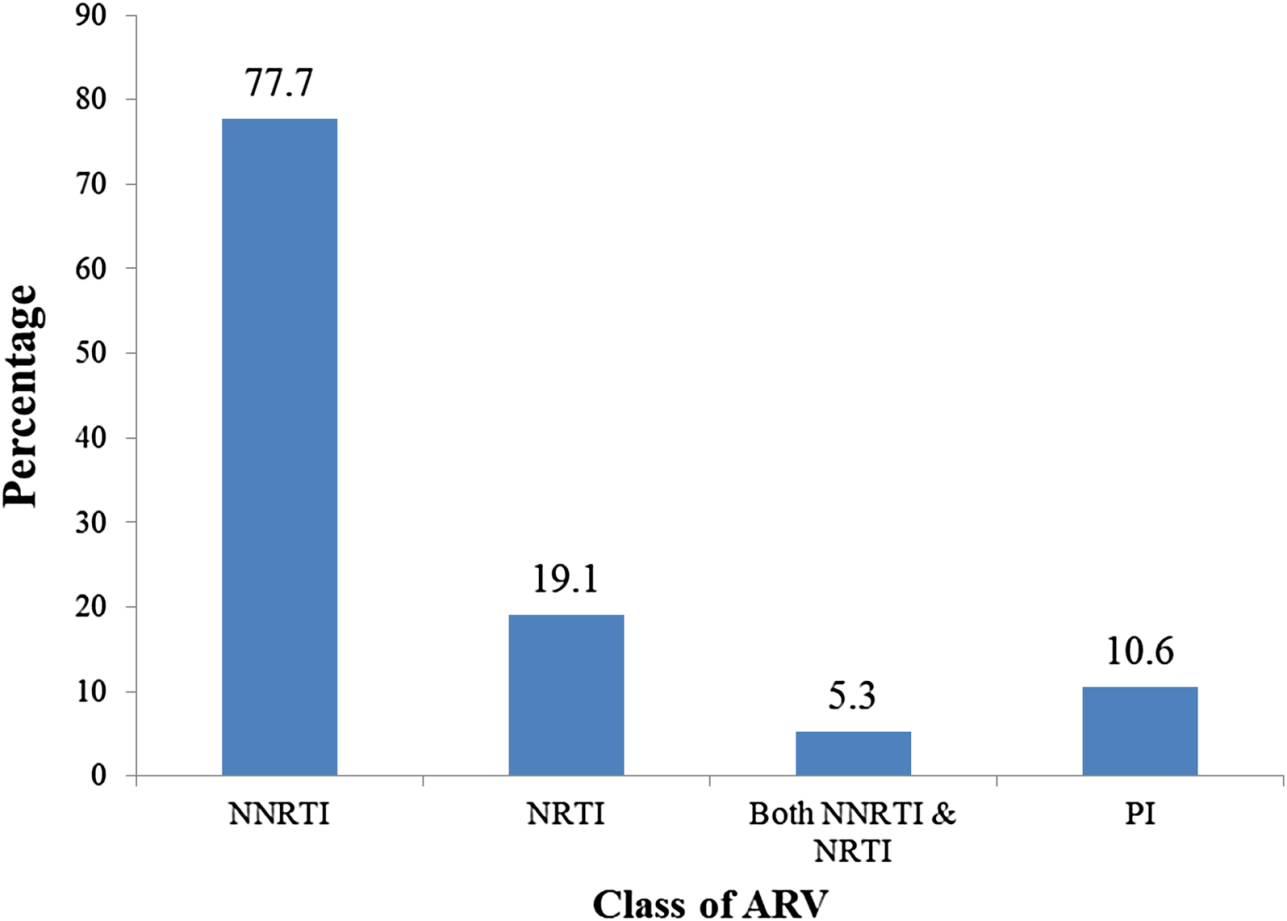
Distribution of baseline antiretroviral drug class mutations among the 93 patients enrolled into the pre-treatment HIV Drug Resistance survey in Zimbabwe: 2008–2010

**Fig. 2:**
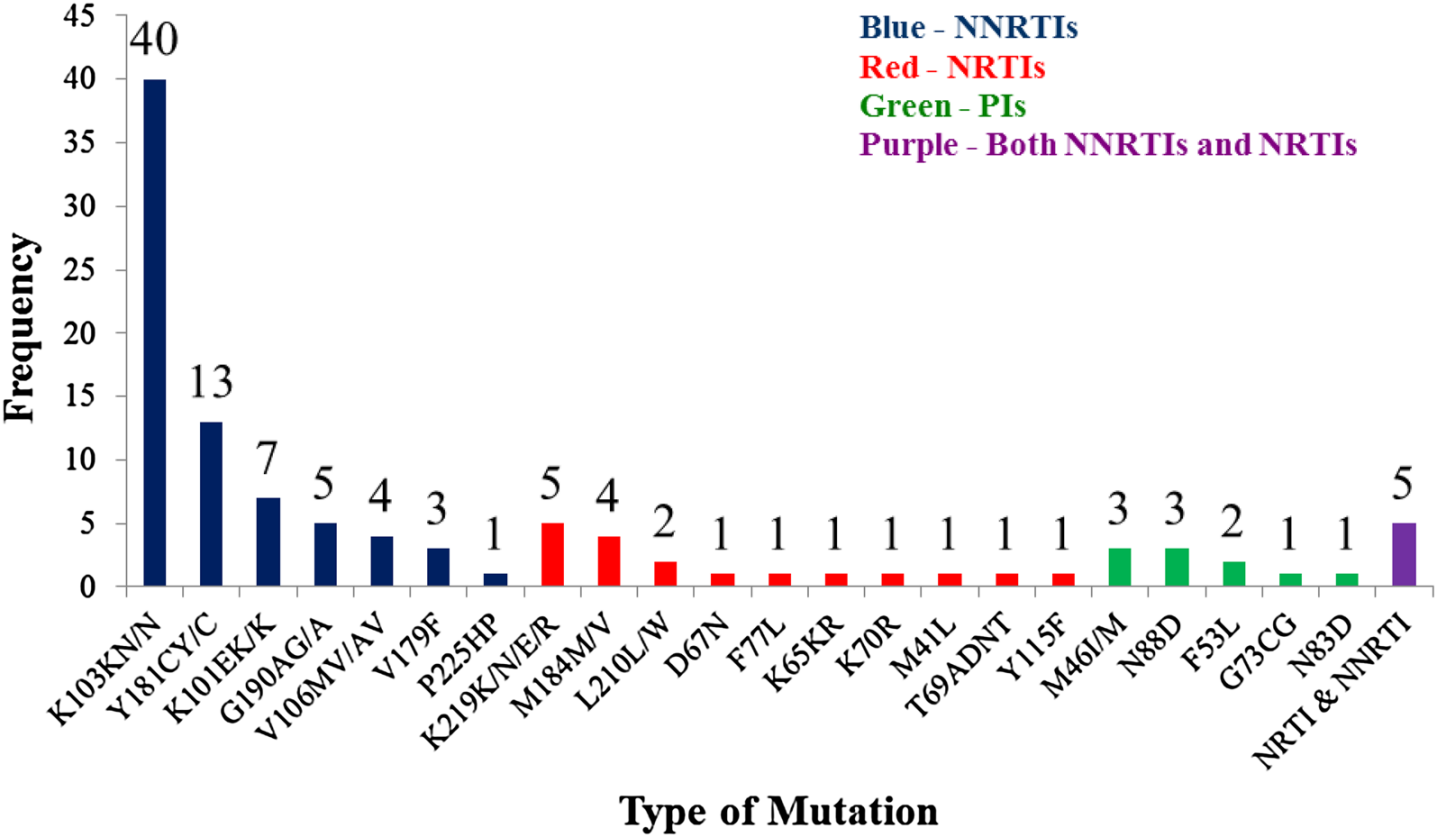
Prevalence of pre-treatment drug resistance mutations among the participants initiating ART and enrolled in the 2008–2010 HIV drug resistance survey

HIV-1 drug resistance genotyping was performed for all 1610 specimens and 92 % of the samples (1483) were successfully genotyped. Among the genotyped participants, 93 [6.3 % (95 % CI 5.2–7.7)] had one or more DR mutations (Figs. 1, 2). The proportion of participants with mutations ranged from 3.0 to 10.3 % at the 12 participating sites. The prevalence of PDR mutations was comparable for men (5.5 %) and women (6.7 %) (*P* = 0.402). The PDR mutations were mainly those against NNRTIs (4.9 %). Among the 73 NNRTI mutations identified, the most common mutations are K103N (2.7 %) and Y181C (0.9 %). These mutations can cause a high level of resistance to efavirenz (EFV) and NVP, the two ARVs that are components of the WHO-recommended first-line regimens. Twenty NRTI mutations were also identified and the most common ones were K219E/N (0.3 %) and M184 V (0.3 %). These mutations also can cause high level of resistance to NRTI drugs: lamivudine (3TC) and emtricitabine (FTC); and a low level of resistance to abacavir (ABC) and didanosine (DDI). The former two ARVs are part of the current first-line regimens recommended by WHO. In Addition, 10 PI mutations were identified and the most common ones were N88D (0.2 %) and M46I (0.2 %) which can lead to resistance to indinavir (IDV), nelfinavir (NFV), lopinavir (LPV) and atazanavir (ATV), in which ritonavir-boosted LPV (LPV/r) and ATV (ATV/r) are the components of the 2nd-line regimens recommended by WHO (Fig. 1).

### Factors associated with PDR

Among those 93 participants with PDR mutations, 10 participants had previous exposure and 65 had no previous exposure to ARVs while 18 had missing data. Prevalence of PDR was significantly higher among those with previous exposure (12.6 %, 95 % CI 5.9–19.8) than those without exposure (5.7 %, 95 % CI 4.0–6.2) to ARVs (*P* = 0.002) and nine of the 10 who had previous exposure were women (Table 4). All three participants who had been exposed to ART had resistance to NNRTI (K103 N) while one had an additional PI mutation (M46I). Similarly, among the 7 women exposed in PMTCT, six had NNRTI and one had PI mutations. Among those exposed to ARVs there was no statistically significant difference in the prevalence of PDR mutations between those exposed to ARVs [16.7 %; CI 5.4–57.2] and those exposed to PMTCT [11 %; CI 4.9–22.9] (*P* = 0.467). Analysis of PDR by CD4 count values (Table 2) showed that 53.8 % had CD4 count below 350 cells/mm^3^. The prevalence of PDR among participants with different CD4 counts was not different (*P* = 0.997).

**Table 2:** Distribution of pre-treatment drug resistance mutations by CD4 count among enrolled patients, 2008–2010

CD4 count (cells/mm^3^)	Drug resistance mutations (N = 1483)	
	With mutation (%)	Without mutation (%)
<200	33 (2.2)	491 (33)
200–349	17 (1.1)	261 (17.6)
≥350	43 (2.9)	635 (42.8)
Missing	0 (0)	3 (0.2)
Total	93 (6.3)	1390 (93.7)

Bivariate analysis showed that only previous exposure to ARVs was significantly associated with PDR (OR 2.58, 95 % CI 1.3–5.12). Other factors which were not significantly associated with PDR were female sex (OR 1.22), WHO clinical stage 3 or 4 (OR 1.14) and CD4 count <350 (OR 1.02) (Tables 3, 4).

**Table 3:** Factors associated with pre-treatment drug resistance mutations among enrolled participants, 2008–2010

Variable	Mutation	No mutation	OR	95 % CI
*Sex*				
Female	68	947	1.22	0.76–1.96
Male	25	426		
*Previous exposure to ARVs*				
Yes	11	65	2.58	1.30–5.12*
No	65	991		
*WHO clinical stage*				
3 or 4	65	887	1.14	0.69–1.88
1 or 2	22	343		
*CD4 count (cells/mm* ^*3*^ *)*				
<350	43	635	1.02	0.6–1.55
≥350	50	752		
*Viral load (copies/ml)*				
≥1000	86	1288	0.47	0.14–1.60
<1000	3	21		

* Statistically significant

**Table 4:** Prevalence of Pre-treatment drug resistance mutations among enrolled participants, 2008–2010

Variable	HIV DR	95 % CIs
*Sex*		
Female	6.7	5.2–8.4
Male	5.5	3.6–8.1
*Previous exposure to any form of antiretroviral drugs*		
Yes	11.6	5.9–19.8
No	5.0	4.0–6.2
*WHO clinical stage*		
3 or 4	6.8	5.3–8.6
1 or 2	6.0	3.8–9.0
*CD4 count (cells/mm* ^*3*^ *)*		
<350	6.3	4.7–8.2
≥350	6.3	4.6–8.2
*Viral load (copies/ml)*		
≥1000	12.5	2.7–32.4
<1000	6.3	5.2–79.1

## Discussion

A moderate level (6.3 %) of PDR was identified among the participants newly initiating ART in the 12 sentinel ART sites in Zimbabwe. The prevalence of PDR was significantly higher among those who had previously exposed to ARVs than those without. Exposure of ARVs and the most previous exposure to ARVs were women enrolled into the PMTCT program. Most participants initiated ART were in the WHO clinical stage III or IV while more than half of all participants had CD4 count below 350 cells/mm^3^. Majority of the participants (95.7 %) had a VL ≥ 1000 copies/ml. The CD4 count, WHO clinical stage, VL, age and sex were not significantly associated with the detection of PDR among those with these variables recorded. In this study, men were more likely to have advanced HIV infection at the initiation of ART than women, despite the likelihood of having PDR being comparable between men and women.

The results of this study are consistent with the findings of a multi-centred observational study in six countries, including Zimbabwe, which showed drug class-specific resistance prevalence of 2.5 % for NRTIs, 3.3 % for NNRTIs, 1.3 % for protease inhibitors, and 1.2 % for dual-class resistance to NRTIs and NNRTIs [5]. In South Africa provincial level studies reported prevalence rates of 4.2 % in Gauteng Province, 2.5 % in Cape Town and 3.6 % in Free State Province between 2002 and 2004 [10]. These values almost mirror what we found in this study. The South African study also suggested rising levels of transmitted drug resistance in the region from 3 % (2005/6) to 7 % (2007/8) [10]. Other studies in different parts of the world have also shown prevalence of HIVDR ranging from 3 to 15 % [3, 11, 12].

In our study, there was no association between age, sex, clinical stage and CD4 count with PDR. Similarly in a study by Hunt et al. [13], there was no association between resistance and CD4 percentage, sex, and WHO clinical stage. However, there was an association with younger age. Therefore, these factors may not be relevant in considering factors associated with having PDR in our population despite the non-random distribution of the study sites.

The resistance mutations described herein were observed after more than 4 years of the availability of treatment in the public health sector in Zimbabwe. It should be noted that, although access to treatment in the public sector began in 2004, ARVs were already available in the private sector, notwithstanding the higher cost, limited access and non-standardized regimens.

The most common PDR mutations detected in the current study were against NNRTIs, and this finding is consistent with the widespread use of this drug class as part of Zimbabwe`s National ART Guidelines as the standard first-line ART regimens, as well as single-dose NVP for PMTCT. As Zimbabwe has adopted the Option B+ strategy where all HIV-positive pregnant women and lactating mothers are commenced on NNRTI-based ART and the finding of an association on detecting PDR with previous ARV exposure mainly through PMTCT in the current study (Tables 3, 4), there is reason to be cautious/alert to the possibility that women/patients having resistance to ARVs prior to initiating the standard first-line ART may be negatively impacted on their treatment outcomes. A study by Hammers and others also showed that 13 % of patients with previous exposure to ARVs had PDR. The authors then proposed still starting patients on the standard first-line regimens with closely monitoring of VL and/or genotyping at 6 months into the therapy [14]. In Zimbabwe this is not possible considering the limited resources, such as laboratory capacity where CD4 count and VL are not even routinely available to patient services.

Irrespective of whether the observed frequency of PDR mutations is predominantly caused by transmission or by selection of undisclosed medication with ARVs, PDR mutations in patients who are starting ART may translate into reduced efficacy of the first-line and second-line ART regimens. As higher ART coverages are achieved as projected, the risk of transmitted HIVDR is likely to be increased.

There are limitations of the current study. Firstly, the sites were not randomly selected to represent all people living with HIV and AIDS in their respective regions. Thus caution is warranted when extrapolating the results to different subpopulations or regions. Secondly, participants were chronically infected and eligible for ART initiation, thus observed PDR may have been transmitted or acquired during earlier undisclosed ART or PMTCT. Thirdly, due to the design of study to enrol chronically HIV infected participants, the PDR prevalence reported here may be underestimated, thus the true prevalence of PDR might be higher than reported here. However, given the challenges of identifying individuals with recent HIV infections in resource-limited countries, there are values in surveying PDR in populations initiating ART in which at least a proxy of PDR in pre-treatment populations can be estimated using national representative sampling method which will provide important information about the probable effectiveness of currently available first-line regimens for each country/region to ensure the effectiveness of national/regional ART programs.

## Conclusions

In conclusion, this cross-sectional PDR survey in ART initiators at 12 purposefully selected sentinel ART sites reveals a moderate level of PDR among the survey participants. To maintain the efficacy of the current first-line regimens there is need to strengthen all HIVDR prevention efforts and to conduct further studies to investigate optimal strategies that can prolong the efficacy of the current first-line ARV regimens in Zimbabwe and beyond.

## Abbreviations

3TC: lamivudine
ABC: abacavir
ART: antiretroviral therapy
ARVs: antiretroviral drugs
ATV: atazanavir
CD4: cluster of differentiation
CDC: Centers for Disease Control and Prevention
CI: confidence interval
DBS: dried blood spot
DDI: didanosine
EDTA: ethylene-diamine-tetra-acetic acid
EFV: efavirenz
FTC: emtricitabine
HIV: human immunodeficiency virus
HIVDR: human immunodeficiency virus drug resistance
LPV: lopinavir
LPV/r: ritonavir-boosted lopinavir
NFV: nelfinavir
NICD: National Institute of Communicable Diseases
NMRL: National Microbiology Reference Laboratory
NNRTI: non-nucleoside reverse transcriptase inhibitors
NRTI: nucleoside reverse transcriptase inhibitors
NVP: nevirapine
OI/ART: opportunistic infection/antiretroviral therapy
OR: odds ratio
PDR: pre-treatment drug resistance
PI: protease inhibitor
PMTCT: prevention of mother-to-child transmission of HIV
RT: reverse transcriptase
SOP: standard operating procedures
VL: viral load
WHO: World Health Organization
